# The Effect of Humidity on Dielectric Properties of PP-Based Nano-Dielectric

**DOI:** 10.3390/ma12091378

**Published:** 2019-04-28

**Authors:** Xiaohong Chi, Wenfeng Liu, Shengtao Li, Xiaohong Zhang

**Affiliations:** 1 State Key Laboratory of Electrical Insulation and Power Equipment, Xi’an Jiaotong University, Xi’an 710049, China; hrbustcxh@xjtu.edu.cn (X.C.); sli@mail.xjtu.edu.cn (S.L.); 2 Key Laboratory of Engineering Dielectric and Its Application, Ministry of Education, Harbin University of Science and Technology, Harbin 150080, China; x_hzhang2002@hrbust.edu.cn

**Keywords:** PP-based nano-dielectric, dielectric properties, damp degradation, water molecules distribution

## Abstract

Nano-dielectrics are sensitive to humidity and easily degraded in damp environment because of the high surface energy of nanoparticles. In order to study the effect of humidity on the dielectric properties of nano-dielectric, polypropylene (PP) was modified by polyolefin elastomer (POE) and nano-SiO_2_, and the samples with obvious filling concentration were pre-selected by breakdown strength for damp aging. The aging experiments were carried out in different relative humidity. The dielectric properties of new, hygroscopic saturation and samples after drying were measured and analyzed. It is found that the breakdown strength of hygroscopic saturation nano-dielectrics decreased obviously compared with new samples, and it was difficult to recover after drying. The damp degradation resulted in different changing trends of permittivity of PP and nano-dielectric, but there were relaxation loss peaks of water in both of them. The influence of damp degradation on the trap distribution was studied by thermally stimulated depolarization currents (TSDC), and it was found that the traps level introduced by water molecules was different in PP and nano-dielectrics. All experiment results showed that the performance of nano-dielectrics degraded obviously in humid environment, and it was difficult to recover even after complete drying because of the existence of bounded water molecules in nano-dielectrics.

## 1. Introduction

Blending nanoparticles is an effective way to improve the dielectric properties of traditional polymer, especially in solving the space charge problems [1,2,3]. In recent years, a large number of high-performance polymer-based nano-dielectrics have been obtained in the laboratory. For example, the filling of SiO_2_ or MgO in polyethylene (PE) and cross-linked polyethylene (XLPE) can effectively inhibit the injection and accumulation of space charge and therefore largely improve the insulation properties of the matrix [4,5,6]. The comprehensive properties of polypropylene (PP)-based nanocomposites have been improved by filling nanoparticles and other means, and it has promising application prospects in insulation structure of thermoplastic cable [7,8,9,10].

However, the application of nano-dielectrics was very limited at present. The main reason lied in the poor stability. In practical, reliability and stability is a key performance index as important as other parameters [11]. With the development of new generation power grid, insulation materials were required to operate in a more complex and harsh environment. For example, the working humidity of power equipment in coastal areas exceeded 70% RH (relative humidity) in long-term, and the insulation of power equipment such as hydropower and offshore wind energy needs to withstand high humid environment for a long time [12,13]. However, the filling of nanoparticles makes the dielectrics more easily degraded in humid environment because of the high surface energy.

Zou et al found that epoxy resin with 3 wt. % nano-SiO_2_ had 40% higher moisture absorption than raw epoxy resin, resulting in two orders of magnitude increase in loss and nearly 10 times increase in electrical current [14,15]. The damping aging experiment of XLPE-based nano-dielectrics at 40% RH showed that the moisture absorption of XLPE/MgO with 3% nanoparticles was 30% higher than that of XLPE, and the conductive current and space charge accumulation of nano-dielectrics increased significantly [16]. The research of Praeger et al. on PE-based nano-dielectrics showed that the polarization characteristics of PE hardly changed after damp aging, and the breakdown strength of PE decreased by less than 5%. However, in PE/SiO_2_ nano-dielectrics, the breakdown strength decreased from 352 kV/mm to 112 kV/mm, and the loss increased by more than two orders of magnitude after damp aging [17]. It could be seen that the moisture absorption of typical polar and non-polar polymer increased with the filling of various filling nanoparticles (SiO_2_, MgO, Al_2_O_3_, etc.), and the dielectric properties of nano-dielectrics decreased more obviously after moisture absorption.

In this paper, nano-dielectrics were prepared by blending PP with the nanoparticle and the elastomer (POE). The samples with better properties were pre-selected by DC breakdown test. Focused on the effect of damp aging on dielectric properties, experiments were carried out under different humidity. The degradation of dielectric properties caused by hygroscopicity was studied by comparing the DC breakdown field strength and polarization of new, damp degradation and drying samples. The distribution of water molecules in dielectrics was analyzed by trap distribution characteristics measurements.

## 2. Materials and Methods

### 2.1. Samples Preparation

**Figure 1:**
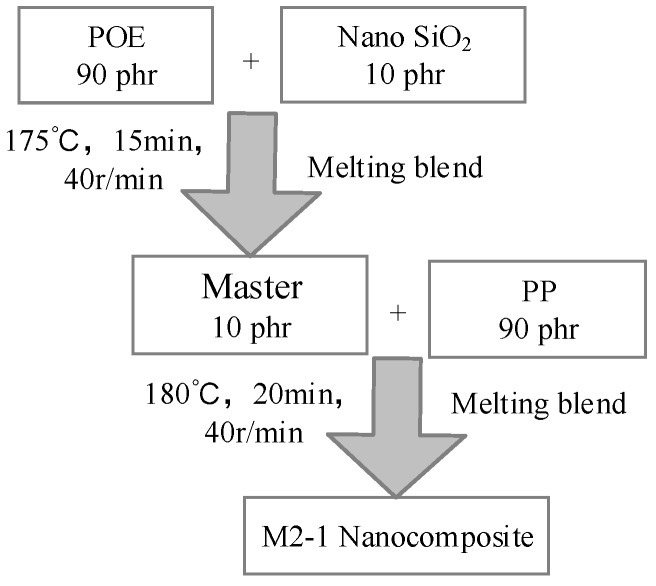
Diagram of two-step melting method, and phr meas parts per hundreds of resin.

The matrix was isotactic PP (HC314BF, Borealis, Abu Dhabi, United Arab Emirates), and the POE (POE-0201, Exxon Mobil Corporation, Shanghai, China) was ethylene-octene copolymer particles with 20% to 30% octene content. The average diameter of nano-SiO_2_ was 30 nm and it was modified by silica agent. The composites compounded of PP and nano-SiO_2_ were melted using internal mixer (HAAKE Polylab QC, torque rheometer, Waltham, MA USA) at 180 °C, counter-rotating at the speed of 40 rpm for 20 min. The composites compounded of PP, POE, and nano-SiO_2_ were prepared by a two-step method. In the two-step method, the nano-SiO_2_ and POE were first melted at 175 °C and 40 rpm for 15 min to prepare the master batch, and then the PP was blended with the master batch at 180 °C and 40 rpm for 20 min to prepare the composite of M2-1. The details of the two-step method is shown in Figure 1.

The abbreviation and component of composites are shown in Table 1. After being melted, samples were pressed into required thickness using compression molding under 190 °C for 15 min.

### 2.2. Breakdown Strength

The DC (direct-current) breakdown experiment was carried out according to the standard of ASTM D1389. The samples were about 0.13 mm in thickness and the sphere–sphere electrode was used in the breakdown experiment. During the test, the electrode and samples were immersed in insulating oil. The applied voltage ramp was 2 kV/s. The Weibull statistic was used for analyzing breakdown properties, so at least thirty valid data should be acquired from each kind of composite. The samples with high breakdown strength were screened by DC Breakdown (BDJC-100kV, Beiguang Precision Instruments Co. Ltd., Beijing, China).

### 2.3. Dispersion of Nano-Filler

The nano-silica dispersion in the samples with high breakdown strength was observed. The POE and amorphous fraction of PP were etched by etch agent, and inner nano-SiO_2_ was exposed. The etch agent was composed of potassium permanganate and concentrated sulfuric acid. The samples were etched in etch agent for 8 h at room temperature before observation. Samples after etching and cleaning were sputtered with gold and observed by scanning electron microscope (SEM, KEYENCEVE-9800S, KEYENCE, Osaka, Japan).

### 2.4. Damp Degradation and Recovery

Samples with high breakdown strength were aged at a steady temperature in a humid box. The degradation temperature was 80 °C. To study the effect of humidity on hygroscopic ratio, the damp aging was performed in the relative humidity of 50%, 75%, and 98% RH, respectively. The hygroscopic ratio of saturated samples was weighed using high-precision electronic balance. The electrical tests were taken with the saturated specimens to study the effect of humidity on the dielectric properties. To research the recovery capability from damp degradation, the dielectric properties of hygroscopic saturated samples were measured again after drying in vacuum at 80 °C for 48 h. New samples, hygroscopic samples and recovered samples were recorded with postfix of -new, -degraded humidity and time, and -renew.

### 2.5. Permittivity and Loss

The frequency dependence of permittivity and loss were measured by broadband dielectric spectrometer (Novo control, Concept 80, Novocontrol GmbH, Montabaur, Germany). Gold electrodes with a diameter of 30 mm were sputtered on the sample before experiment. The test was performed at 20 °C with the frequency ranging from 0.1 Hz to 1 MHz.

### 2.6. Relaxation Processes

Thermally stimulated depolarization currents (TSDC) were measured to analyze the relaxation processes. The sample with sputtered gold electrodes (Φ 30 mm) was used in the TSDC measurement. Firstly, the sample was polarized at 100 °C under the electric field of 2 kV/mm, and then the temperature was rapidly dropped to −50 °C. After that, the polarized voltage was removed and the depolarization current was recorded when the temperature rose from −50 °C to 150 °C at the heating rate of 3 °C/min.

## 3. Results and Discussion

### 3.1. Sample Pre-Selected by Breakdown Strength

**Figure 2:**
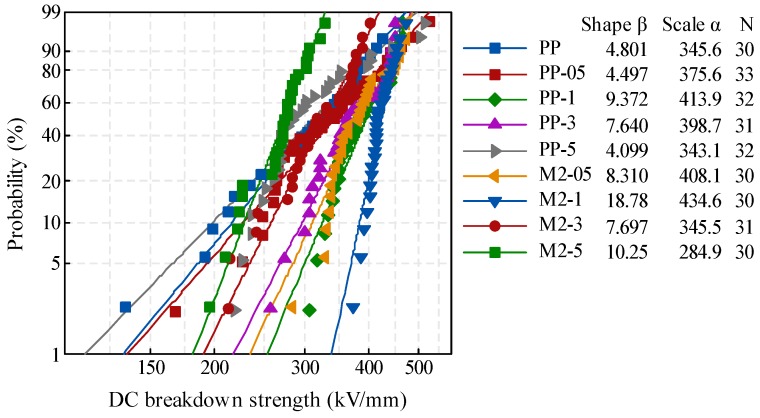
Weibull distribution of DC breakdown strength.

The breakdown strength was an important parameter to evaluate the insulation reliability of the material. In this study, the DC breakdown strength of composites with different content of filler was measured to pre-select firstly. The failure of dielectric followed a certain probability, and the Weibull distribution was usually used to analyze the breakdown data [18,19]. The scale parameter α, which is known as the characteristic breakdown strength (kV/mm), is the strength at which the breakdown probability is 63.2%. The shape parameter β represented the dispersion of breakdown data. The Weibull distribution of breakdown strength results were shown in Figure 2.

In the composites compounded of PP and nano-SiO_2_ (PP-05 to PP-5), the breakdown strength was firstly increased and then decreased as filler content increased. And the highest breakdown strength occurred in samples containing 1 phr nano-SiO_2_.The introduction of POE could modify the mechanical properties of PP, but the continuous distribution of POE formed a weak area for electric field concentration, and therefore reduced the breakdown strength [20]. Nevertheless, in the composites composed of PP, POE, and nano-SiO_2_ (M2-05 to M2-5), the maximum value of breakdown was obtained in M2-1, which contained 1 phr nano-SiO_2_ and 10 phr POE. Compared with pure PP, the α increased by 25% and β raised about four times in M2-1. The breakdown results showed that the PP-1 and M2-1 could be pre-selected as the potential insulation of HVDC cables, so the damp degradation and dielectric properties of these two samples were analyzed.

### 3.2. Dispersion of Nano-Silica

**Figure 3:**
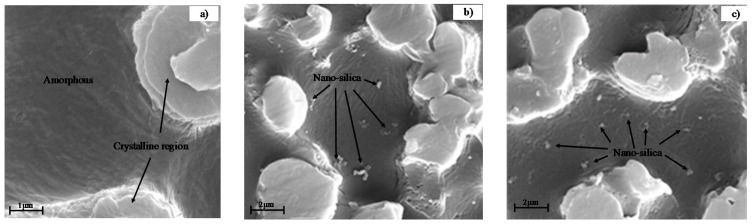
Dispersion of nano-silica particles in composites. The salient and continuous region was crystal fraction of polypropylene (PP) that was not attacked in etching treatment, and the small spherical region was nano-silica. (**a**) PP, (**b**) PP-1, and (**c**) M2-1.

The SEM images of selected composites are shown in Figure 3. After POE and amorphous PP were etched by an etch agent, the crystalline region became salient and the nano-silica was exposed. The small spherical region was nano-silica, and it dispersed evenly in PP-1 and M2-1. The interaction between nano-silica and polymer was different in PP-1 and M2-1. The nanoparticles of PP-1 were protruded, while the nanoparticles were embedded in the matrix in M2-1.

### 3.3. Hygroscopic Ratio in Degradation

**Figure 4:**
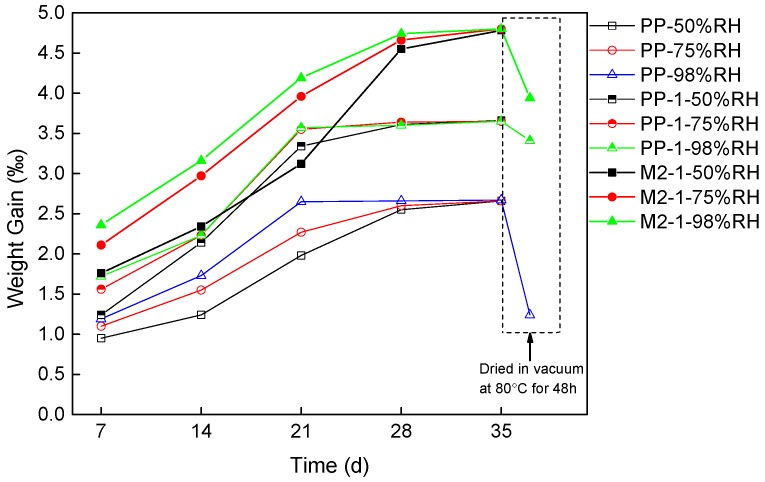
Hygroscopic ratio change of PP, PP-1, and M2-1 degraded at 50%, 75%, and 98% RH for 35 days, and after dried in vacuum at 80 °C for 48 h.

The samples of PP-1 and M2-1 with high breakdown strength were degraded in damp environment, and PP was set as a control group. The temperature was kept at 80 °C, and the damp degradation experiments were carried out under the relative humidity of 50%, 75%, and 98% RH. The hygroscopic ratio of saturated samples was weighed with high-precision electronic balance. The variation of samples’ weight gain over time is shown in Figure 4. The hygroscopicity of the samples for the first 28 days (d) had a positive correlation to humidity. The hygroscopicity weight gain of the samples under low humidity was lower than that under high humidity condition. With the prolongation of degradation time, the hygroscopicity did not further increase and finally reached saturation. After 35 days of degradation, the hygroscopicity of all samples in different humidity environments reached saturation. The hygroscopicity saturation of each type of samples was basically the same in different humidity conditions, indicating that humidity would only affect the time it takes to reach saturation but would not affect the final hygroscopicity saturation state.

Comparing the saturation of different types of samples, the hygroscopicity of composites at all degradation stages was higher than that of pure PP. The weight gain of moisture was lower than 2.5‰ in PP while higher than 3‰ in the composites. This result showed that nanoparticles enhanced the hygroscopicity. This may be due to the fact that nanoparticles are hydrophilic and of strong polarity. Although some hydrophilic groups could be replaced after surface organic treatment, the surrounding area of nanoparticles was still the main area where water molecules adhere to. Therefore, the moisture absorption rate of the composites with nanoparticles was higher than that of pure PP. The composite with POE and nanoparticles absorbed more moisture than other two groups. This can be attributed to that more void for moisture was provided in the amorphous POE around nanoparticles. After dried in vacuum at 80 °C for 48 h, some of the free water would migrate out. Hence, the gain weight of all samples decreased after dried treatment. However, there still remained more gain weight in composites than that in pure PP. The different changes of gain weight between pure PP and PP with nanoparticles were sourced from the water molecular in bound state.

### 3.4. Effect of Hygroscopicity on Breakdown

To study the influence of moisture absorption on dielectric strength, the DC breakdown experiment was carried out before and after degradation. The characteristic field strength of breakdown was calculated from Weibull distribution. The results of unaged, saturated and dried samples were shown separately in Table 2. After damp degradation, the breakdown strength of PP, PP-1, and M2-1 decreased to 74%, 38%, and 33.3% of the original strength. After dried in vacuum at 80 °C, the breakdown strength of these three kinds of specimens recovered to 84.5%, 64.1%, and 56.7% of the original strength, respectively. In conclusions, the experiment results showed that more hygroscopicity of composites would lead to a significant decrease in DC breakdown strength, which could not be restored to its original state after vacuum drying. It is no doubt that this effect has a strong negative impact on the engineering application of nanocomposite dielectrics.

After moisture absorption was saturated, the breakdown strength of composites decreased by more than 60%. This was because it was easier to absorb more water molecules around the nanoparticles, and the composites were also easier to absorb moisture. Water molecules around nanoparticles formed structural defects and electric stress concentration, resulting in a significant lowering of breakdown strength. After drying, part of the free water would migrate away, and the breakdown strength would be restored accordingly. However, the breakdown strength of composites was difficult to recover to the original level. Besides, it was lower than that of PP after drying. The main reason was that water molecules in composites were bounded by nanoparticles and difficult to migrate during drying [21].

### 3.5. Effect of Hygroscopicity on Permittivity and Loss

**Figure 5:**
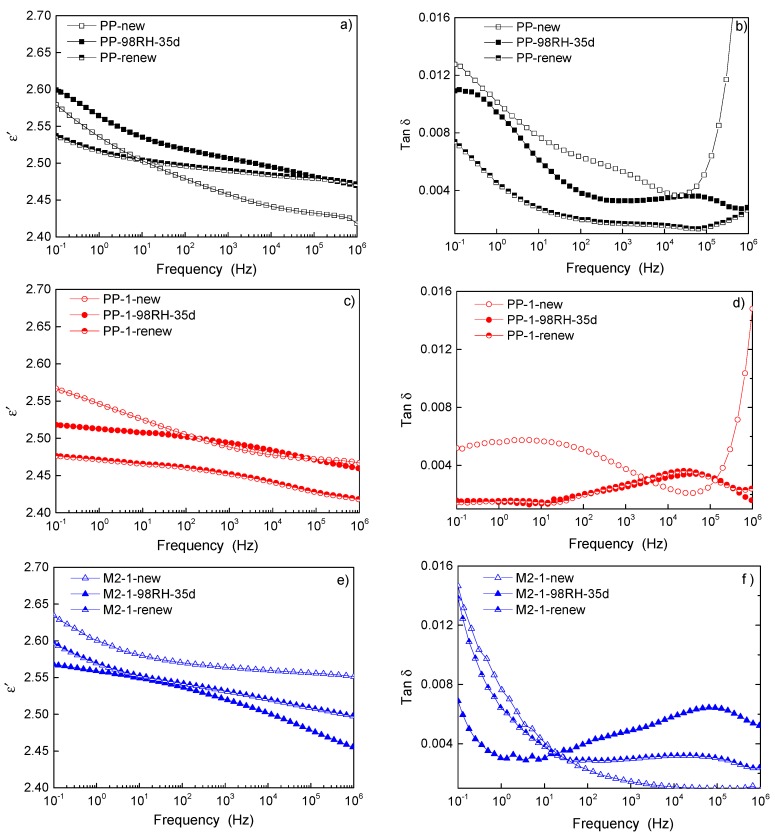
The permittivity and loss of new, degraded, and recovered samples. (**a**,**b**) The real permittivity and loss of new, degraded, and recovered PP. (**c**,**d**) The real permittivity and loss of new, degraded and recovered PP-1. (**e**,**f**) The real permittivity and loss of new, degraded, and recovered M2-1.

Water molecules distributed in damp samples not only affected the breakdown strength under strong electric field, but also affected the polarization response under weak field. The variation of real part of permittivity and loss with frequency are shown in Figure 5. Figure 5a,c,e showed the real permittivity of PP, PP-1, and M2-1, respectively, at different degradation stages. In the frequency range from 0.1 Hz to 1 MHz, hygroscopicity led to the increase of permittivity in PP, and it recovered somewhat after drying. Unlike pure PP, the permittivity of nano-dielectrics decreased after damp degradation. In addition, the decrease of permittivity is more obvious in M2-1 than PP-1. After drying treatment, the permittivity of these two samples was still lower than that of the new sample. It could be seen that the opposite change of permittivity in PP and nanocomposites resulted from damp degradation, which was related to the interaction between water molecules and samples.

Figure 5b,d,f showed the frequency-depended loss of PP, PP-1, and M2-1, respectively, at different degradation stages. The hygroscopicity reduced the loss at frequency less than 10 Hz in the three sample groups, because water molecules hindered the directional movement of carriers under the test field strength. The obvious relaxation loss peaks appeared in the degraded samples at frequency range from 104 Hz to 105 Hz, indicating that the water molecules in the samples formed polar groups and relaxation polarization loss under alternating electric field. After vacuum drying, the relaxation loss peak caused by hygroscopicity in PP disappeared, while that in nanocomposites persisted. This showed that water molecules were distributed freely in PP matrix while bounded in nanocomposites [22]. Vacuum drying could only remove part of free water molecules, but it was difficult to remove bound water molecules. As the polarization and conduction processes were only related to the structure itself in new samples, the permittivity was correlated with a loss in the new PP and its nanocomposites. After damp aging, the water molecules dispersed and interacted with samples, which affected the conductivity and relaxation process. Therefore, the dependence of permittivity and loss with frequency became complex in damp samples.

From the real permittivity, it could be seen that the relaxation time of different samples varied, which was related to the secondary ordered structure formed around the nanoparticles [23]. For the same sample, the relaxation time did not change significantly before and after damp aging. Because the sample was non-polar, the relaxation loss was small, and the conductivity changed obviously due to moisture absorption. It was difficult to obtain the relaxation loss characteristics by superimposing the relaxation loss and conductivity loss in loss diagram. The relaxation time of the new relaxation loss peaks was relatively concentrated, which was attributed to the relaxation polarization of water molecules.

## 4. Discussion

**Figure 6:**
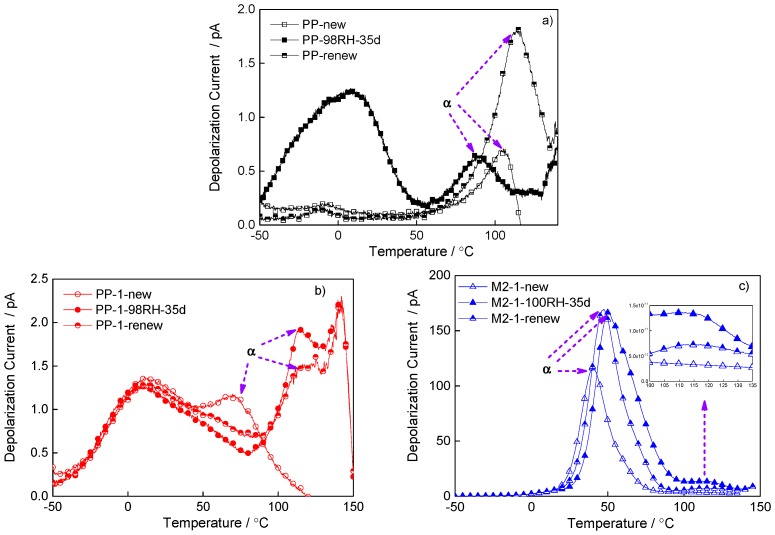
The depolarization current of new, degraded, and recovered samples. (**a**) Depolarization current PP; (**b**) depolarization current PP-1; and (**c**) depolarization current of M2-1.

To further explore the mechanism of damp degradation-induced changes in dielectric properties, the TSDC was employed to show the influence of water molecules on trap distribution. The trap distribution of polymer insulation, which affected the charge distribution and dielectric properties, was related to material structure, impurity, and defect distribution. The trap distribution could be represented by the depolarization current formed by charges released at different temperatures. The depolarization current of PP and the nanocomposite were shown in Figure 6. The α peak in depolarization current was corresponded to the charges release related to molecular relaxation of crystalline structure in PP [24]. The damp degradation affected the release of charges related to the movement of molecular chains in crystallization, so the α peak in the damp sample moved towards the low temperature, and the charges released after the drying treatment was still large. An obvious depolarization current peak appeared in the damp PP between −50 °C and 50 °C. This was due to a large number of shallow traps formed because of water molecules. These shallow traps captured a large number of charges in the process of polarization, and depolarization current peaks occurred in the process of depolarization. The current peaks appeared at low temperature because the free water was obviously affected by temperature [25]. After drying treatment, this peak disappeared as the free water was removed.

Compared with pure PP, damp degradation resulted in a shift of the α peak toward high temperature in PP-1, indicating that the distribution of water molecules in nanocomposite was different from that in pure PP. As the strong interaction was formed between water molecules and nanoparticles, the depolarization current peak from the existence of water appeared above 100 °C. The water molecules were bounded around nanoparticles, and they had obvious thermal movement when the temperature was high [26]. The charge captured by the bound water molecules would collapse at high temperature and form depolarization current peak. After drying treatment, the current peak introduced by damp aging persisted, which means that it was difficult to remove the bound water in nanocomposite during drying process.

The blend of amorphous POE shifted the molecular chain movement to low temperature, and the depolarization current released by the chain movement at a lower temperature accordingly, as shown in Figure 6c. Similar to that of PP-1, damp aging resulted in the shift of the α peak toward high temperature due to the addition of nanoparticles in M2-1. The depolarization current peak from water molecules also appeared above 100 °C, and the peak did not disappear after drying treatment.

By comparing the depolarization current characteristics of new, damp, and renewed samples, it could be seen that the main state of water molecules in pure PP sample was free water molecules, and could be removed after drying. The appearance of depolarization current peaks in low temperature in PP showed that shallow traps are mainly introduced into by free water molecules. The shallow traps led to the decrease of DC breakdown strength and affected polarization characteristics. In nano-dielectric samples of PP-1 and M2-1, water molecules were bounded by nanoparticles, and it was difficult to remove them by drying at 80 °C. The interaction between bound water molecules and nanoparticles introduced deep traps, and those traps could capture charges at low field, so the internal electric field was homogenized and the real permittivity of nanocomposites was reduced. However, unlike the deep traps introduced by nanoparticles, the deep traps introduced by bound water had negative effects on short-term breakdown. This was because under strong electric field, the existence of water in the damp sample will form local defects, leading to electric field stress concentration and accelerating breakdown.

## 5. Conclusions

The PP-based composites with various POE and nano-SiO_2_ content were fabricated by melting blend method, and samples were pre-selected by DC breakdown strength. The high breakdown strength appeared in PP-1 which the nano-SiO_2_ content was 1 phr, and the M2-1 with 10 phr POE and 1 phr nano-SiO_2_ also had high breakdown strength. The damp degradation was performed on the optimum samples of PP-1 and M2-1, and compared with the pure PP as control group.

Comparing the saturation of different types of samples, the relative humidity would only affect the time it takes to reach saturation, but would not affect the final hygroscopicity saturation. The hygroscopicity of composites at all degradation stages was higher than that of pure PP, and it was difficult to remove the bounded water formed in nanocomposites.

The damp degradation affected the dielectric properties of nano-dielectrics obviously. The DC breakdown strength of nano-dielectrics decreased by more than 60%, and it was difficult to recover after drying. The hygroscopicity resulted in different changing trends of permittivity of PP and nano-dielectric, but there were relaxation loss peaks of water in both of them. Different from PP, the real permittivity of the nanocomposites decreased after hygroscopicity, and it was lower than that of the new sample after drying.

The TSDC results provided the further proof of the different states of water in pure PP and nano-dielectrics. The appearance of depolarization current peaks in low temperature in PP showed that shallow traps are mainly introduced into by free water molecules. In nanocomposite samples of PP-1 and M2-1, the interaction between bound water molecules and nanoparticles introduced deep traps, and those traps could capture charges at low field, so the internal electric field was homogenized and the real permittivity of nanocomposites was reduced.

## Figures and Tables

**Table 1:** Abbreviation and component of samples.

No.	Mass Fraction, phr			Description
	PP	POE	Nano-SiO_2_	
PP	100	0	0	Raw PP, as a control group
PP-05	100	0	0.5	Composites compounded of PP and nano-SiO_2_; the number suffix denotes the content of nanoparticles
PP-1	100	0	1	
PP-3	100	0	3	
PP-5	100	0	5	
M2-05	95	5	0.5	Composites compounded of PP, POE, and nano-SiO_2_; the number suffix denotes the content of nanoparticles
M2-1	90	10	1	
M2-3	70	30	3	
M2-5	50	50	5	

**Table 2:** The characteristic strength of breakdown got from new, degraded, and recovered samples (kV/mm).

Condition	PP	PP-1	M2-1
New	345.6	413.9	434.6
Degraded under 98% RH and 80 °C for 35 days	255.8	157.4	144.9
After degraded, dried in vacuum at 80 °C for 48 h	291.9	265.3	246.5
